# Feedback in family medicine clerkships: a qualitative interview study of stakeholders in community-based teaching

**DOI:** 10.1080/10872981.2022.2077687

**Published:** 2022-05-18

**Authors:** Roland Koch, Julia Braun, Stefanie Joos

**Affiliations:** Universitätsklinikum Tübingen, Institute for General Practice and Interprofessional Health Care, Tübingen, Germany

**Keywords:** Undergraduate medical education, dialogic feedback, community-based learning environments, family practice, qualitative research, quality management

## Abstract

Feedback is an important aspect of teaching and learning in medical education. Irrespective of the training environment, too little effective dialogic feedback occurs. Community-based outpatient learning environments, such as general practitioner practices, have heterogeneous framework conditions regarding feedback that decrease feedback quality. To improve feedback in this setting, characteristics of feedback in such learning environments must be considered. This study aims to reveal such characteristics from different perspectives and derive ideas for improving feedback in community-based learning environments. Three stakeholder groups in family medicine clerkships as an example of community-based learning environments (n = 15 students, n = 12 faculty and administrative staff, n = 13 general physician trainers) were interviewed for this study. Transcripts of the interviews were analysed with qualitative content analysis. All stakeholders interviewed note a lack of feedback between groups. Feedback in primary care practices takes place in specific contexts (e.g., during vs after a consultation, during vs at the end of the clerkship) and is provided in different ways (e.g., verbal vs nonverbal). Barriers of effective feedback in community-based settings are: lack of opportunity/initiation, fear of giving feedback, unawareness (of correct feedback and/or lack of prior experience with feedback), and little basis for feedback. Currently, the exchange between the university and community-based learning environments is limited to grading and report writing, with little sharing of meaningful information. The potential of a better exchange between those within community-based learning environments and the university to improve feedback processes is not reached. This exchange and the framework conditions specific for the community-based learning environment should be considered as parts of the structural dimension of feedback. Teachers and course managers of family medicine institutes are in an important position to shape these factors actively, working together with stakeholders of community-based teaching.

## Introduction

Feedback is a facilitator of medical students’ competency and professional development [1–4]. The ability to give and receive feedback has been defined as a core competency objective for German medical students in the National Competency-Based Learning Objective Catalogue (NKLM) [5]. The feedback process comprises a communication loop in which the medical student (‘learner’) performs a task (e.g., a vaccination) which is observed by a peer or a teacher (e.g., a general practitioner (GP), ‘trainer’) [6]. The trainer evaluates the task verbally to improve the learner’s future performance in the observed task [7–9]. Optimal feedback should be timely and should be based on direct observation [10,11]. Adequate preparation of learners and trainers, the alignment with learning objectives, and optimal feedback technique contribute to successful feedback [8,11]. The emotional impact of feedback must be considered, and a ‘safe space’ for feedback should be provided [11].

Recent studies have introduced the idea of feedback as a bidirectional or dialogical process, in which both participants act as senders and receivers [1,12,13]. Yang and Carless identified that such dialogical feedback has three dimensions: The abovementioned feedback content, a social-affective dimension in which feedback is negotiated between sender and receiver and a structural dimension, which corresponds to the organisation and environment in which feedback takes place [14]. The authors identified all three dimensions as viable targets for feedback improvement efforts.

In clinical education, feedback environments include on-campus learning sites (e.g., in the university hospital) and community-based learning sites (in private primary care practices or community hospitals). Such learning sites are referred to as decentral or community-based environments [15,16]. In Germany, the two-week family medicine clerkship and the four-week clinical elective take place in GP practices associated with the medical faculty [15,17–19]. GP practices are independent of the university, and the GPs are not employed as university faculty. Learning experiences in such environments offer unique opportunities for individual supervision and feedback [8]. A previous study revealed that GP-student feedback occurs in only 31% of the patient consultations performed by students in GP practices [20]. External conditions, such as lack of time, often prevent optimal feedback in GP practices [10,21–23]. Feedback processes are further influenced by the one-on-one teaching relationship and the interaction between trainer and learner [10,21].

In 2025, the medical licensure act will be reformed [1]. One of the reasons for this reform is a relative shortage of physicians willing to work in community-based outpatient settings. With this reform, the existing medical curriculum will expand to community-based learning environments on a much larger scale than today. Little is known on how course managers at the university could best prepare trainers and students in community-based outpatient settings for optimal feedback [20,21,24].

The aim of this study is to reveal and describe feedback barriers and enablers specific to the GP practice learning environment from the trainers’, learners’ and faculty’s perspectives. The study aims further to derive ideas for preparing trainers and learners for optimal dialogical feedback in this environment.

## Method

The study was conducted as a cross-sectional qualitative interview study at Tübingen University between 2017 and 2018. It follows the constructivist paradigm and draws on Kolb’s theory of experiential learning, which sees learning as a process of generating knowledge and skills through the transformation of experience. The process comprises transactions between learners and the learning environment [25]. Results are presented adhering to the SRQR reporting guidelines for qualitative research [26].

### Setting

Tübingen University Hospital is located in southern Germany. The Department of family medicine at the medical faculty cooperates with around 250 family medicine teaching practices. About 160 students complete a clinical elective in one of these practices during the first two weeks of each semester. The teaching practices are located in communities within a radius of up to 70 km around the city of Tübingen.

### Participants

The study comprised three groups of stakeholders involved in teaching and learning in family medicine clerkships. The groups were:

1. Students (sixth-year medical students at Tübingen University)
2. Trainers (GP trainers in the community)
3. Faculty and administrative staff involved in the organisation of the family medicine clerkship from different medical faculties in Germany (Teaching department managers/heads of department or medical and non-medical teaching coordinators). Representatives from this group will be called ‘faculty’ in this article.

Concerning the participants of this exploratory study, a wide range of experiences and opinions with a narrow focus (teaching in family practice) was to be obtained. With this in mind, Malterud’s concept of ‘information power’ was applied, and a sample size of 15 interview participants for each group was estimated [27]. Participants were initially approached by email. Individuals who sent a positive response to the email were included in study on a first-come, first-served basis if they met the inclusion criteria. Trainers and students were additionally approached in person during teaching seminars. Eligible participants (see Table 1) that gave written informed consent were interviewed by JB between 2017 and 2018 either via telephone or in-person.

**Table 1.** Inclusion and exclusion criteria

Group	Inclusion criteria	Exclusion criteria
1 (Students)	family medicine clerkship completed at the medical faculty of Tübingen University (sixth study year)	family medicine clerkship not yet completed (study years 1–5) family medicine clerkship not completed at the medical faculty of Tübingen University
2 (GP Trainers)	GP physicians associated with and accredited by Tübingen University Prior supervision of medical students in the family medicine clerkship or the Practical year (PJ) in family medicine	Had not yet supervised students in the family medicine clerkship or the Practical year (PJ) in family medicine, former teacher/mentor to the interviewer, other relationship that might cause a bias
3 (Faculty)	Member of a German medical faculty in family medicine involved in the organisation of the family medicine clerkship	Supervisor of the present study, Another member of the same university already took part in the study, Interview partner is not involved in the organisation of the family medicine clerkship

## Data collection

A separate interview outline was developed for each group based on the existing literature and observations of family medicine clerkships at the medical faculty of Tübingen University. The questions explored learners’, trainers’ and faculty’s views and experiences on teaching and learning in GP practices. Particular emphasis was placed on the role of feedback. No definition of feedback was given to the interview participants. Additionally, the gender and age of each participant were asked. The translated interview guidelines are attached as supplementary file 1.

Interviews were audio-recorded and transcribed verbatim. Any identifying information was removed from names and places. No repeat interviews were conducted.

### Qualitative content analysis

Qualitative content analysis was applied to the interview transcripts of all three interview groups [28], using the software f4 analyse (dr. dresing & pehl GmbH, Marburg, Germany). The analysis started in 2018 and was finished in 2019. One coding frame for all interviews was developed as follows:

An initial coding frame was built by JB. Interview transcripts were inductively separated into units of meaning. These were then grouped deductively using the major topics of the interview guidelines [28]. RK and JB then used this initial coding frame to double-code one interview in each stakeholder group separately. They then compared their results. Ambiguities and differences that arose, for example, through applying the coding frame to a particular group, were discussed. The coding frame was specified and supplemented so that the codes were applicable to all stakeholder groups [29]. One additional new interview was double-coded by RK and JB to ensure consistency, followed by another discussion and refinement of the coding frame. This consensus coding frame was then used by JB to code all interviews.

Finally, the coding frame’s main and subcategories were screened by JB for quotes and topics related to feedback. Transactions between stakeholder groups (written, oral or nonverbal communication, sharing experiences) with the intention to affect or assess the learning process in GP practices was considered feedback [25]. This was followed by an analysis of the excerpts aimed at describing barriers and enablers of dialogic feedback in GP practices from the integrated perspective of all those involved in community-based teaching.

### Researcher characteristics and self-reflection

RK is a family physician, education researcher and head of teaching at the Institute of General Practice and Interprofessional Health Care at the Tübingen University. He is responsible for the organization of the family medicine clerkship, the recruitment of GPs, the didactic training of GP trainers, and the management of problems during feedback. SJ is a family physician and head of the department. She is responsible for family practice funding and for contracts with practices. SJ and RK have regularly been involved in teaching, both in community family practice and at the university. JB is a physician in training in family medicine and a graduate of Tübingen University. She had personal experience with community-based feedback as a medical student. Thus, she had such experiences in common with the students interviewed. Furthermore, a few of the interviewed students were known to JB. The fact that she was a recent medical student helped to create a trusting atmosphere and closeness in the interviews. It also meant that JB’s background differed significantly from the interviewed GPs and faculty, which may have resulted in more distance in those interviews [30]. Some of the interviewed faculty and GP trainers were known to RK and SJ, but the transcripts were pseudonymized before analysis. The authors would like for more dialogic feedback to occur in community-based teaching.

## Results

Forty interviews with a duration between 8 and 45 minutes were conducted, n = 15 (37.5%) as in-person interviews, with the remainder as telephone interviews. Demographic characteristics of interview participants are presented in Table 2. An excerpt of the coding frame with corresponding interview quotes is shown in Table 3.

**Table 2.** Participant demographics

Group	1 (Students)(n = 15)	2 (GPs)(n = 13)	3 (Faculty)(n = 12)
Age (Median; Min-Max)	26 (23–32)	60.5 (38–68)	50 (33–62)
Missing (n% total)	0 (0%)	1 (7.7%)	1 (8.3%)
Female (n% total)	12 (80%)	4 (30.8%)	6 (50%)

**Table 3.** Excerpt of the coding frame, main category ‘feedback’

CATEGORY	SUBCATEGORY		CITATION
Content	technique (e.g., observed procedures)		*‘And then to hear from someone with experience: “Okay, […] the suspected diagnoses you thought of were right, but we’ll start by checking this and that.” So I think this kind of case discussion I find most helpful because that just helps me find the right thread.’ (ST-1, 41)*
	medical issues		*I: “Did you receive helpful feedback from your teaching GP?“ ST: “Yes, […] especially on how to interact with the patients, because I didn’t really know how to deal with them. Am I allowed to be frank and direct, or do I have to hold back[…] And they always told me: ‘No, you did it all well, it’s okay like that.’ That was great.“(ST-7, 21)* *GP: “and I mean feedback goes both ways … I once … I once, how should I put this now … actually treated a patient unmedically because he was very difficult, and the student pointed it out to me by asking, questioning what I was doing now and then I realised, ‘Actually, he’s right!’“ (GP-2, 68)*
	behaviour, appearance, attitudes		*General Practitioner (GP): „[…] And one day I asked him: ‘Listen! Why did you actually choose to study medicine?’ And then he said his marks had been good enough and a doctor had a good reputation and would earn money. And then I told him: ‘Boy, […] that’s not enough for this job as you will experience so many things where reputation and money are not enough. And the reputation, you don’t get it with the title, you have to earn it. I: “How did he react?” GP:’ Nicely! […] He thought about it and then the next day he came and asked […]:’How did you mean that?’ And then I told him certain things.” (GP-711-13 and 113–114)* *‘I: Do you give your students feedback regarding their behaviour? B: I always do that … […] that’s very important, that someone gets feedback, whether he now seems too timid, or anxious, or in a certain situation perhaps would have had other options.’ (GP-5, 32)*
	didactics and method		*I:” […] did you give feedback to your teaching physician […]? ST: Yes. So when I was allowed to do something, like taking a blood sample from the jugular. I already told him that I thought it was good that I was allowed to do that because otherwise you never have the opportunity. Or that you are only allowed to do that after watching once. I also thanked him, for example, when I was allowed to be present during medication-assisted treatment or was allowed to interview patients.” (ST-7, 27)”* *GP: „But at the end of the clerkship, I always ask if there is something that should be changed fundamentally, or what they were missing […] Unfortunately, so far, they did not mention anything they were missing, so I don’t know if they like it or not. (GP 11, 60–61)*
Frame	Situation	Fb talk at the end of the clerkship	*ST: ‘So at the end of the clerkship, he [The GP teacher] showed me how he filled out the evaluation form, and consequently I guess he was satisfied.’ (ST-11, 28)”*
		Fix timeslots for fb during the clerkship	*‘Every day between 10 and half past 10 […] every morning there is a conversation if we have time for it. [.] If there is still something to discuss from the previous day, whatever comes to mind.’ (GP-12, 39)*
		Fb during consultations	*ST: „He presumed a lot of knowledge. Because I could not remember everything at once, there were comments that stated that I should know this … this sometimes happened while a patient was present.” I: ”Ok, and how did that feel?“ ST:‘Well, not that great.’ (ST-2, 29–30)*
		Fb talk in a special setting outside the practice	*GP: ‘Typically, I invite [students] I’ve grown fond of on the last evening of the clerkship. There is a nice restaurant nearby, and we go there for dinner, and I tell a little about what has moved me [during the clerkship], what was special or especially different … what was good and what was not that good.’ (GP-5, 36)*
	Initiation	GP initiates feedback	*GP: ‘And what I additionally do is: I ask them after the first half of the time, well they start on Tuesday[.] and on Friday they get the task against the background of what they have already experienced to think about: What is still missing? What do I have to do in the following week?’(GP-7, 61)*
		Student is seeking feedback	*ST: „And then in the end, I explicitly asked [my teaching GP] … I don’t know if otherwise that would have come from her part. I asked her if she had any tips for me, what I should improve, or revise or do […]”(ST-4, 25)*
		Spontaneously given feedback	*ST: ”After every patient [encounter], he closed the door and briefly discussed the case with me and told me what I could have done differently. “ (ST-15, 31)*
Indirect feedback	Nonverbal feedback		*ST: „Sometimes [the GP] glanced at me questioningly when I wasn’t able to do something because I only knew it in theory or probably had forgotten a lot of things.” (ST-2, 23)*
	Self-reflection, feedback through (success) experiences		*ST: My most positive experience [during the clerkship] was that I made a diagnosis correctly, which actually was not too easy. It was about atypical pneumonia. I thought by myself: This sounds strange down there to the right[…] … and as the patient came back with the X-ray, there was really something to see on the imaging, and I was really proud of myself.” (ST-3, 40)* *GP: ‘Occasionally I do tell [the students] if they do something good, I do give feedback, and I think they notice it as well as they are allowed to do more things and work more and more independently. (GP-10, 26–27)’*
Problems	Feedback does not take place	lack of opportunity/initiation	*I: ‘Did you get useful feedback from your teaching GP?’ ST: „Not too much … . Actually, this didn’t really happen […] the script for the clerkship says that a feedback talk should take place … we didn’t really do that … actually we did not do it at all.” (ST-1, 26)* *I: “Did a student ever give you feedback on your work as doctor and as teacher? GP: ‘Well, that’s what I’m actually missing a bit. I get feedback if I ask for it, if I ask the students in the end: What was good, what was bad? What would you say was great? The students usually give very little [feedback], and I’d wish for more. Even negative aspects: “I’ve noticed you do this that way and at university we learned it differently. Why do you do it like that?” I’d like to get more feedback and would be happy to receive it.’ (GP-12, 41)*
		(Not) daring to give feedback	*ST:”I didn’t pass criticism on him, no […] I don’t know if it would have been understandable for him, because […] it’s a lot of subjective feeling, as well. (ST-2, 33)* *GP: ‘[…] one feels that the students are not frank [with the teaching GP]. I think they are just afraid to say something wrong. Even though you stress that they can’t say anything wrong … . I think as a student, you just don’t say it.’(GP-11, 59)*
		Unawareness	*ST: I have to admit at that point in time I wasn’t really aware of it. […] Before, I had not experienced [that it could be different], and I could not say that it was bad. […]” (ST-6, 56)* *GP: No, I didn’t report [the problematic behaviour of the student [to the faculty], as it was one of my first, or the first student, and I was rather surprised how little she took the clerkship seriously. Only over time, I had the comparison, how conscientiously all the others dealt with the clerkship.” (GP-10, 38)*
		little basis for feedback	*ST: ‘Well, I would not have known what I should have been given feedback for […] of course I would have liked to receive more [feedback] but that would have implied that I could have done more on my own.’ (ST-6, 44–45)*
ASSESSMENT AND EVALUATION	Student grading	Student assessment sheet	*F: ‘For one thing, on the evaluation sheet that’s in our accompanying booklet, the teaching physicians can add a comment at the bottom. “Very involved, very interested, disinterested, shouldn’t be in general practice, would be better off in research, can’t hold conversations … ” that, of course, is reflected in the grading.’ (F-10, 51)*
		Case report	*F: „That means I’m relying on what the students write to me on my request. Of course, I prompt them to give written feedback, but I probably get five feedback reports in one semester with 160 students. Most of them are formulated very generally, like: „Was great!” or something like that. And probably one feedback per semester, by rule of thumb one feedback per semester contains specific feedback.” (F-9, 19–20)*
	Information forwarding		*F: ‘A teaching practice doesn’t get the evaluation of one student directly. Only if the teaching practice had more than five students for the clerkship, the evaluations of this group are handed down directly to the teaching practice. […]That’s how the rules are.[…] We are regularly informed by the teaching practices, that this is not effective, because they either get no direct feedback at all …, of course, they get information of the department on how the clerkship went in general […]but no [direct feedback] for the respective practices. They don’t like that, but we do not currently have another solution.’ (F-8, 24–27)* *‘I: In what way do you receive feedback from the institute for your teaching activities […]? B: We don’t really receive any feedback. I don’t know. At least specific. Sometimes you find out something on a “GP day” [CME for GPs at campus]. There used to be an evaluation of the students’ evaluation forms. But that was then always general, […] and about one semester, so nothing specific.’ (GP-1,63)*

The coding frame consisted of four main categories (‘teaching method’, ‘feedback’, ‘teacher-student interaction’, ‘structure and organisation of the family medicine clerkship’). The main category ‘feedback’ with its subcategories is presented in the table below with appropriate quotations and excerpts from the interviews. The citations were translated from German to English by the authors. Text citations refer to the stakeholder group (ST = student, GP = General Practitioner, F = faculty) and paragraph: E.g.,: ST-15, 31 reads as student interview 15, paragraph 31. The interviewer is abbreviated with ‘I’.

The following sections elaborate on the above overview.

### Feedback content

GP trainers provided feedback on medical or diagnostic issues. This included pointing out gaps in knowledge, providing feedback on work ethics, medical procedures, examination techniques, and the students’ clinical reasoning. They gave treatment suggestions and commented on students’ interactions with patients. Feedback from the family physicians to the students also related to student behaviour. This included the students’ appearance and demeanour. The students’ attitudes toward the medical profession and general practice were also the subject of feedback.

Feedback from students to their respective teaching physicians related to observations on medical procedures and decision-making, therapeutic or organizational procedures in the practice, and didactic methods.

## Feedback frame

### Situation

Both students and GPs reported a variety of situations in which GPs provided feedback to students. While some GPs gave feedback spontaneously, others deliberately created structured feedback opportunities such as a scheduled time slot or short breaks between consultations. Several interviewees mentioned that feedback was given mainly at the end of the clerkship during a final talk. Some GPs reported having this feedback talk outside the practice environment, e.g., in a restaurant.

Feedback was provided both in the presence of a patient and in a 1:1 encounter between trainer and student. According to the interviewees, feedback in the presence of the patient offered the advantage that it could be implemented immediately. At the same time, the feedback recipient could perceive the situation as awkward.

### Initiation

Participants described that feedback was initiated most often by the teaching GPs. However, they also reported situations in which students requested feedback proactively. In most cases, GPs had to ask for feedback from the learner to the trainer specifically.

## Indirect feedback

Student participants reported non-verbal communication by the GP trainer, such as nodding in agreement or raising an eyebrow, which was perceived as feedback. They also reported reflecting on learning experiences and discussing them with peers. Primarily positive learning experiences were described as a form of indirect feedback.

Teaching physicians reported successively entrusting students with more independent activities during the family medicine clerkship if they performed well. These activities provided learning opportunities and were described as a form of feedback.

## Feedback problems

Students’ assessments of the feedback received varied. Some students were satisfied with the amount and kind of feedback, while others complained about a lack thereof. GPs expressed the wish for more feedback as well. From the perspective of the interview participants, the main problem was that feedback did not take place at all. Four specific reasons for the perceived lack of feedback were named:

### Missing opportunity and initiation

Students and GP trainers often reported that feedback did not happen at all, and both sides explicitly wished the other to initiate feedback more often. If not explicitly initiated by the trainer (see above, Initiation) or the learner, an opportunity for feedback was wasted.

### Not daring to give feedback

Students were reported to avoid expressing criticism openly, which often prevented them from providing feedback to trainers. Both GP trainers and students assumed that feedback recipients might be hurt or would not understand the criticism. These assumptions, as well as the hierarchy and power dynamics between students and GPs, were described as barriers to feedback. If the family physician explicitly signalled openness to feedback and there was a sustainable relationship between GP trainers and students, feedback from students to the GP was more likely to occur. Avoiding feedback instead led to uncertainty and the abovementioned desire for more constructive communication on both sides.

### Unawareness

Another cause mentioned by the interview participants for the limited feedback is unawareness about feedback. Without having comparable experiences or awareness of effective feedback, one might find it difficult to formulate what could be improved or what should be different. This problem was described on the student as well as the GP side.

### Little basis for feedback

Another aspect mentioned by the students is that a lack of feedback is often a consequence of missing opportunities to perform tasks independently: If you are not allowed to do anything, you cannot be observed and given feedback.

## Assessment and evaluation

### Student grading

At the medical faculty of Tübingen University, teaching physicians reported evaluating their students using a standardised evaluation form. In addition, grading was sometimes used as a feedback opportunity.

According to faculty, a student evaluation sheet is used at most university sites. The grading information was then relayed to the central family medicine university departments. GP trainers sometimes included additional information or comments about students on the grading sheets. Sometimes additional evaluation formats, such as evaluation telephone calls, were used. These evaluations were traceable to the respective teaching practice as case reports. However, faculty reported a lack of feedback from student evaluations and stated that student evaluations often reflected only the extremes and did not provide representative insights.

### Information forwarding

Several faculty reported that the data protection policies of the university restricted the transfer of non-anonymous student evaluation to the teaching GPs. Feedback from students written on evaluation sheets thus did not reach the teaching GPs or did so only with a substantial time delay. Most of the teaching GPs indicated that they received no feedback whatsoever from universities for their teaching. GPs pointed out that a lack of such feedback hindered their efforts to improve their teaching.

## Integrated analysis: feedback pathways in family medicine clerkships

Most feedback occurred in community-based remote locations. It was initiated mainly by GP trainers. Unidirectional feedback from the trainers to the student was prevalent. Bidirectional or dialogical feedback was reported less frequently, especially if learners took the initiative and became active.

I: Would you have liked more feedback overall? B: I am someone … if I want to know something, then I also ask for it, so from that point of view, that was exactly right. I can’t say now exactly how much of it came from me and how much from him, but so all in all, it was good. I: Okay, that is, sometimes you actively asked for it and sometimes it came directly from him. B: Yes.” (ST-3, 20)

**Figure 1.**
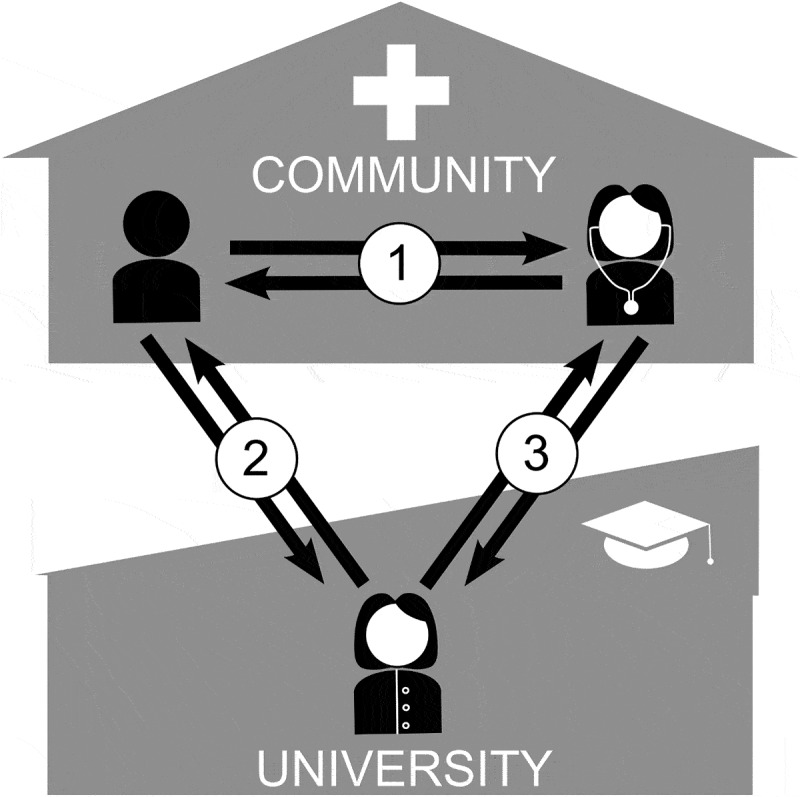
Communication pathways in community-based family medicine clerkships; 1: On – site between teacher and learner; 2: between learner and faculty (e.g., case reports); 3: between community-based teacher and university (e.g., student assessment sheet).

Integrating the stakeholders’ descriptions of feedback revealed three bidirectional communication pathways in which feedback could occur either directly or indirectly: Feedback between 1) learners and trainers, 2) learners and faculty (directly and through evaluation and grading), and 3) trainers and faculty. While the first process occurred in community-based learning environments such as GP practices, the latter involved family medicine departments at the university and thus represented communication between community- and university-based teaching. It put family medicine departments in a position to relay feedback between students and preceptors. Figure 1 visualises this.

## Discussion

Qualitative interviews with stakeholders involved in family medicine clerkships revealed that feedback in GP practices is complex, and a third party (faculty) needs to be considered. Several barriers and enablers to dialogic feedback in GP practices were identified, reflecting the heterogeneity of teaching practices and stakeholder perspectives.

### Feedback barriers in the GP practice setting

The present study shows that too often, too little feedback is initiated from the perspective of all participants, which explains the lack of feedback in GP practices identified by Bösner et al [20]. Likewise, the participants established a connection between feedback and learning opportunities and experiences. The lack of feedback is perceived as a barrier to optimal learning [1,12].

Using Yang and Carless’ model, some barriers can be attributed to social-affective dimension [14]: While the trainer has the primary responsibility for feedback initiation, the lack of initiative on the student’s side also contributes to the lack of feedback. Some of the identified barriers for feedback can be found in the existing literature, like power dynamics [10], preconceptions [24,31], as well as difficulties in giving feedback on behavioural aspects [1,12]. This study also shows that a lack of prior experience and, consequently, a lack of comparison with one’s own or others’ learning experiences prevents feedback. Additionally, our data show that the lack of feedback to trainers also hinders their instructional development.

When feedback occurs, its quality depends on several factors. As postulated before, the lack of independent, supervised learning opportunities prevents timely and specific feedback [4,10]. Furthermore, if feedback is delivered in clinical situations and in front of patients, a regular occurrence in GP practices due to the high volume of patient visits, the preceptor needs to consider the specifics of the situation [32]: timely feedback given in front of patients is not necessarily within a ‘safe environment’ [4,11,33]. Words must be chosen with care and mindful of the circumstances. While most previous studies focussed on verbal feedback [1], the present study shows that even nonverbal expressions are perceived as feedback and can unsettle the learner.

Late or untimely feedback, even if well-intentioned, can prevent the trainer from observing and re-evaluating recommended changes in the learner. Moreover, the later in the clerkship feedback is given, the more it is mistaken as a summative assessment, which hurts feedback quality due to the power dynamics mentioned above [34]. As long as learners fear consequences as a result of their feedback, they will be reluctant to provide feedback to trainers [33]. At the same time, students are unlikely to initiate feedback early in the clerkship because they do not feel comfortable yet.

GP practices as an example for community-based learning environments thus offer complex challenges for high-quality feedback. However, the extent to which the opportunities for helpful feedback are used also depends on trainers and learners being aware of their rights and responsibilities and making use of existing support structures (e.g., involving the university) [14].

### Feedback enablers in the GP practice setting

Examples of successful feedback given by the interview participants generally meet the known requirements of being timely, specific, and in a safe environment [1,4,11]. Several opportunities for structured feedback are identified: between consultations, at the end of the day, during lunchtime or as scheduled appointments. If time is actively allotted for such feedback opportunities, they will likely occur [1,4,35].

Concerning the power dynamics, the interview participants’ experiences show that if a GP trainer explicitly initiates feedback, demonstrates skill in giving feedback, and signals openness towards receiving feedback, he or she encourages the student to give feedback as well. A trusting relationship between trainer and learner facilitates difficult feedback topics such as behaviour, attitudes, or professionalism [8,10].

In accordance with previous studies, our results show that observations made during supervised learning experiences are the basis for feedback [4,10]. Supervised learning experiences provide many opportunities for timely feedback and encourage learners’ self-reflection [36]. Even if previous studies demonstrated downsides of self-assessment, such as self-observation bias [37], the present study shows that learner’s self-assessment is a common form of indirect feedback in GP practices. This may be due to specifics of the GP practice setting, which requires learners to perform independently. As Johnson et al. stated, the encouragement of self-reflection is a facilitator of feedback [9]. Reasonable steps would be for trainers to explicitly label the provision of opportunities for independent learning experiences as feedback, e.g.,”I trust you to be more independent because you did a good job in this situation … .” and asking students to be more self-reflective, e.g., ‘Tell me what you think about your performance in this situation.’

### How can a dialogic feedback process in GP practices be promoted?

Creating more feedback opportunities and transforming feedback from a ‘delivery process’ to a ‘dialogic process’ would drastically improve the quality and quantity of feedback, especially in the long run [38]. Presently, however, this transformation is slowed down mainly by unawareness of all involved stakeholders about the problem [20], lack of training in feedback processes, and specificities of learning environments outside of the university campus, such as travel distances, independence from the university, and differences in scope and clinical mission [16]. Again referring to Yang and Carless’ model, these obstacles affect the structural dimension of feedback in the family medicine clerkship as two different and independent organisations (university and GP practices) are involved [14]. The following section will discuss why Family medicine departments are uniquely positioned to promote the feedback pathway in community-based learning environments and how to address the structural dimension of dialogic feedback.

Both students and trainers should be empowered to actively create an optimal feedback exchange in the GP practice [12,39]. Clear information about what kind of feedback is suited and how optimal feedback is delivered should be provided to both trainers and learners [11]. Teachers and learners should be given the opportunity to experience optimal feedback, e.g., in a seminar. They should also be informed about the goals of the clerkship and didactic requirements of the teaching practice beforehand by the family medicine department. This is in line with the findings of Jamshidian et al., who identified a lack of knowledge about ‘desirable performance criteria’ as a barrier to adequate feedback [40]. Transparent reports on learning experiences of previous student cohorts could provide a reference frame for the next cohort of students in the family medicine clerkship. Quotations from the present study, or fictitious examples based on it, could serve as source material. The impact of student experiences on feedback, feedback as a learning experience and a part of the learning process in the broader context of learning in medical education seems insufficiently understood and will be the focus of future research by our group.

Additionally, structured opportunities for exchange should be created [11]. A scheduled midpoint meeting among students and teaching GPs after the first half of the clerkship could be implemented as part of the clerkship curriculum. If feedback proves difficult in certain trainer-learner pairings, faculty could act as mediators for feedback processes. To prevent long-distance journeys for the participants, such meetings could be held via remote online services. The possibility for feedback after the elective in the form of student evaluation must be communicated to the intended recipients. A digital event reporting and evaluation system, as designed by Fleit et al. [41], could provide additional opportunities for feedback. Further investigations will be needed to evaluate the effects of such interventions.

The before-mentioned tasks are best initiated and moderated by faculty, such as course coordinators at family medicine departments. They play a significant role in expanding teaching capacity and are often the first point of contact for new teaching practices with little experience in feedback delivery. Coordinators must be sensitive to the importance of on-site feedback. Overall feedback quality and quantity can be improved by carefully moderating a transformation from rare feedback towards dialogical feedback. In contrast to previous feedback models, the presented case would not be a feedback dialogue exclusively between trainer and learner but a trialogue (compared with Figure 1): departments act as mediators and conveyers of information until dialogic feedback processes have been fully established in community-based learning environments. They would also receive valuable feedback on their organisational processes.

## Strengths and limitations

To maintain maximum openness towards unforeseen results and not restrict our field of vision with the survey instrument, a qualitative design and an exploratory, inductive approach to analysis were chosen for this study. Representatives of stakeholders involved in family medicine clerkships were included to consider the topic from various relevant perspectives. Sociodemographic characteristics leaned more towards experiences of female students, while the GP group had more experiences of male, middle-aged trainers. Both demographics were consistent with the group of trainers and students, but this might change in the future due to more female GPs becoming trainers.

In terms of generalizability, this study has limitations due to the number of respondents per group. However, interviewees complemented each other by giving examples about participants in the other group (e.g., a preceptor talking about a student), which resulted in a breadth of experiences on the topic of feedback in line with the explorative study design.

The coding framework was developed by two authors. The same authors also coded two interviews per group to develop the coding frame. While this increases the chance of bias, two relevant perspectives (recent medical graduate and faculty) were represented in the process. Optimally, a GP trainer should have been involved in the coding but this was not possible due to financial and time restrictions.

The omission of a unified definition of feedback for the interviews caused most interview partners to interpret the term ‘feedback’ based on their personal experience, attitudes and professional context. This generated heterogeneous responses that leaned more towards feedback content (GP trainers), the socio-affective dimension (students and GP trainers), and the structural dimension (faculty). We accepted more variance between respondents due to the explorative nature of the study. A learning point and limitation was that each interview participant’s own definition of feedback should have been included in the interview guideline to be considered in the analysis.

Interviews with faculty were conducted nationwide. While their opinions are underrepresented in the presentation of our results due to the focus on the main actors in teaching and learning – trainers and students – faculty provided insights into what is happening outside of Tübingen. They highlighted similar challenges and case studies across Germany and provided insights into the structural dimension of feedback elsewhere. Their reports thus provided a degree of external validation for the study.

## Conclusion

Feedback in decentral or community-based learning environments such as family medicine clerkships involves three bidirectional communication pathways: The most important pathway for learning is on-site between trainer and learner. However, there are communication pathways between learner and faculty and preceptor and faculty as well. The latter two pathways involve both the community-based learning environment and a university representative. Feedback between medical students and GPs shows challenges in the cognitive and socio-affective dimension of dialogic feedback: lack of opportunity or initiation, power dynamics, lack of comparison and lack of supervised clinical work. At the same time, timely and effective feedback does occur, especially if the GP trainer provides supervised learning opportunities, demonstrates openness to receiving as well as skill in giving feedback and promotes self-reflection. Both learners and trainers wish for more dialogic feedback. To implement dialogic feedback processes in GP practices, the structural dimension of feedback must be considered. Our research identified two other communication pathways that bridge the gap between university and community-based training. They could provide a means to address the structural dimension of feedback. Family medicine departments could serve as a feedback mediator or a third participant in the feedback dialogue, transforming the feedback dialogue into a feedback trialogue and facilitating on-site feedback in community-based teaching.

### Summary of conclusions

**Table:** 

Four main reasons contribute to the lack of feedback in community-based teaching: Lack of initiation and opportunities, not daring to give feedback, unawareness (of correct feedback and/or previous experiences with feedback), and little basis for feedback. In community-based teaching environments, feedback between students and GP trainers is supplemented by two additional yet unused communication pathways that involve university faculty as feedback facilitators Universities are therefore in a unique position to facilitate the implementation of effective feedback in community-based learning environments Both instructors and learners should be prepared to give and receive effective feedback, and university faculty should be aware of their role as feedback facilitators

## Supplementary Material

Supplemental Material

## Data Availability

The datasets generated and/or analysed during the current study are not publicly available to protect interview partners but are available from the corresponding author on reasonable request.
