# Supplementary material for: Feedback in family medicine clerkships: a qualitative interview study of stakeholders in community-based teaching
Source: Med Educ Online. 2022 May 18;27(1):2077687. doi: 10.1080/10872981.2022.2077687 (PMC9122355; doi:10.1080/10872981.2022.2077687)
Supplement: Supplemental Material [file ZMEO_A_2077687_SM5305.docx]

# Interview questions for medical students

- - - - Start with presentation of the interviewer and context of the interview
- Presentation of the respondent(s): Name, What semester

A. Initial questions (warm-up)

- What is your overall impression of the general medicine block internship?

B. Main questions

- How would you describe the behavior of your teacher towards you?
- Were there any behaviors on the part of your teacher that you felt were problematic? How did you react to them?
- Have you ever felt badly treated? How do you define bad treatment in this context?
- Were there moments when you wished you had ended up in another practice? If so, why?
- Was there a problem with which you turned to the Institute of General Medicine?
- Did you receive helpful feedback from your teaching physician? In what context (during/between consultations, feedback discussion)?
  - Would you have liked more?
  - Did you request feedback?
  - What kind of feedback did you find most useful?
- Did you give feedback to your teacher? In what context?

C. Wrap-Up

- What did you still want to say or have always wanted to say in this context?
- Did we forget anything?

# Interview questions for GP trainers

- - - - Start with presentation of the interviewer and context of the interview
- Presentation of the respondent(s): Name, place of residence since when and where family doctor, since when teaching doctor, how many trainees supervised?

A. Initial questions (warm-up)

- When you heard about the topic of this study, was there a specific situation you remembered?
- Why did you decide to participate in the interview?

B. Main questions

- How do you go about teaching the students in the block internship? Have you changed anything about your approach since you became a teacher?
- What do you think are the prerequisites on the part of the students that are necessary for the students to be successful in learning? What are beneficial and what are obstructive attitudes of the students?
- What do you consider problematic behaviour on the part of the students? Have you had experiences with inappropriate behaviour of the students towards you or towards the other members of the practice team or towards patients?
  - Possible follow-up questions: In which situation? How did you deal with it? How did you proceed from there?
- Do you give feedback to your student regarding his/her behaviour (professional appearance, social competence, communication skills, (body) language)?
- In what context and in what form (structure, time, space) do you give this feedback?
- Has a student ever given you feedback on your work as a doctor and/or teacher or asked for something specifically (possible examples: feedback, guidance, freedom...)?
  - Possible follow-up questions: In which situation? How did you deal with it? How did you proceed from there?
- Were there incidents where you gave feedback to the Institute of General Medicine?
- In what way do you receive feedback from the Institute for your teaching activities? Would you like more?

C. Wrap-Up

- What did you still want to say or have always wanted to say in this context?
- Did we forget anything?

# Interview questions for Experts

- - - - Start with presentation of the interviewer and context of the interview
- Presentation of the respondent(s): Name, position at faculty, since when, how many trainees / students supervised?

A. Initial questions (warm-up)

- How are the teaching practices at your university accredited? Is it difficult to find enough teaching practices?
- What kind of feedback on teaching practices does your institute receive from students?

B. Main questions

- Do "problem practices" surface? How would you define them? How do you deal with them? Are there any consequences for misconduct?
- What is the structure for avoiding "problem practices"?
- In what situations have students sought to change teaching practice? How did they deal with this as an institute?
- What kind of feedback on students does your institute receive from the teaching staff?
- How do teaching practices deal with problematic behaviour of students?
- How does the institute deal with students who have attracted attention through problematic behaviour? Are there consequences (training, disciplinary measures)?

C. Wrap-Up

- What did you still want to say or have always wanted to say in this context?
- Did we forget anything?
